# Novel Poly(L-lactide-co-**ε**-caprolactone) Matrices Obtained with the Use of Zr[Acac]_**4**_ as Nontoxic Initiator for Long-Term Release of Immunosuppressive Drugs

**DOI:** 10.1155/2013/607351

**Published:** 2013-10-28

**Authors:** Katarzyna Jelonek, Janusz Kasperczyk, Suming Li, Piotr Dobrzynski, Henryk Janeczek, Bozena Jarzabek

**Affiliations:** ^1^Centre of Polymer and Carbon Materials, Polish Academy of Sciences, M. Curie Sklodowskiej 34, 41-819 Zabrze, Poland; ^2^Department of Biopharmacy, School of Pharmacy, Medical University of Silesia, Narcyzow 1, 41-200 Sosnowiec, Poland; ^3^Institut Europeen des Membranes, UMR CNRS 5635, Universite Montpellier 2, Place Eugene Bataillon, 34095 Montpellier, France; ^4^Jan Dlugosz University in Czestochowa, Institute of Chemistry, Environmental Protection and Biotechnology, Armii Krajowej 13, 42-100 Czestochowa, Poland

## Abstract

Slowly degradable copolymers of L-lactide and **ε**-caprolactone can provide long-term delivery and may be interesting as alternative release systems of cyclosporine A (CyA) and rapamycin (sirolimus), in which available dosage forms cause a lot of side effects. The aim of this study was to obtain slowly degradable matrices containing immunosuppressive drug from PLACL initiated by nontoxic Zr[Acac]_4_. Three kinds of poly(L-lactide-co-**ε**-caprolactone) (PLACL) matrices with different copolymer chain microstructure were used to compare the release process of cyclosporine A and rapamycine. The influence of copolymer chain microstructure on drug release rate and profile was also analyzed. The determined parameters could be used to tailor drug release by synthesis of demanded polymeric drug carrier. The studied copolymers were characterized at the beginning and during the degradation process of the polymeric matrices by NMR spectroscopy, GPC (gel permeation chromatography), and DSC (differential scanning calorimetry). Different drug release profiles have been observed from each kind of copolymer. The correlation between drug release process and changes of copolymer microstructure during degradation process was noticed. It was determined that different copolymer composition (e.g., lower amount of caprolactone units) does not have to influence the drug release, but even small changes in copolymer randomness affect this process.

## 1. Introduction

Synthetic biodegradable polyesters constitute a large group of materials, commonly used in tissue engineering, as medical devices and in controlled drug delivery systems for producing various implantable and injectable drug carriers [1, 2]. Copolymers of L-lactide and *ε*-caprolactone (PLACL), as slowly degradable materials, can provide long-term delivery and present great interest for immunosuppressive drugs [3]. Poly(*ε*-caprolactone) characterizes good biocompatibility, biodegradability, and permeation to drugs [4]. Materials obtained from poly(*ε*-caprolactone) PCL undergo slower degradation than polylactide (PLA), poly(lactide-co-glycolide) PLAGA, and poly(D,L-lactide) PDLA [5, 6] and may be used in systems, which provide drug delivery even extending over a period of more than one year. As a material appropriate for long-term applications, PCL was considered in controlled delivery of levonorgestrel as Capronor [7, 8]. Copolymers of lactide and *ε*-caprolactone are synthesized for better efficiency of degradation rate, permeability, and thermal and mechanical features that improve their processing and range of applications [6]. They can be also interesting in developing alternative release systems of cyclosporine A (CyA) and rapamycine (sirolimus), in which available dosage forms cause a lot of side effects. Different kinds of CyA carriers obtained from homo- and copolymers are based on lactide and *ε*-caprolactone [7–12]. There were also studies on release of rapamycine from biodegradable matrices (e.g., made of poly(D,L-lactide) and poly(D,L-lactide-co-glycolide)), but carriers obtained from copolymers of lactide and *ε*-caprolactone have not been analyzed yet [13]. Moreover, most studies concerning this agent are related to rapamycine eluting stents, because it has been shown to inhibit growth of vascular smooth muscle cells [14]. 

Besides, there is the necessity of new polyesters development with particular physical and mechanical features, because of dynamic progress in designing and application of novel drug carriers. It was suggested that a wider range of materials need to be screened in the aim of obtaining system of controlled CyA delivery with satisfactory release rate [10]. However, apart from macrostructure, selection of copolymers with appropriate microstructure is also important, because it influences directly physical, thermal, and mechanical features [15], which, subsequently, affect degradation rate and drug release profile. Tailoring the degradation rate of the drug carrier is critical for controlled release of bioactive agents [16]. Mechanical properties and degradation rate can be controlled in a wide range by modification of polymer composition and chain microstructure. Modifications of copolymer chain microstructure can be obtained by changing reaction conditions as time, temperature or kind of initiator [17]. The use of zirconium (IV) acetylacetonate [Zr(Acac)_4_] as a non-toxic initiator of polymerization allows obtaining high-molecular weight copolymers with good mechanical properties [18]. Two modes of transesterification, susceptible to change chain microstructure of the final polyester, could occur during copolymerization of L-lactide and *ε*-caprolactone. The first mode consists of intermolecular exchange of lactidyl (LL) units or their multiplets. Transesterification of the second mode involves bond cleavage in the lactidyl units, leading to lactyl (L)-OCH(CH_3_)CO– sequences [17]. In this case, the polymer chain contains three kinds of sequences: LL-lactidyl–(O–CHCH_3_–CO–O–CHCH_3_–CO–)–; L-lactyl OCH(CH_3_)CO; Cap-caproyl–(O–CH_2_–CH_2_–CH_2_–CH_2_–CH_2_–CO–)–. The influence of lactidyl blocks in the copolymer chains on the release rate of progesterone and *β*-estradiol was described by Buntner et al. More even release rate was observed in case of copolymer obtained from D,L-lactide compared to copolymers synthesized from L-lactide [19]. Among four P(D,L-lactide-co-*ε*-caprolactone) copolymers with different comonomers ratio, the most even release profile was obtained for copolymer containing 83–93% of D,L-lactide [20]. Analysis of three types of poly(L-lactide-co-trimethylene carbonates) (PLATMC)—two semiblock and one random, revealed that copolymer microstructure influences also the release of immunosuppressive drugs (cyclosporine A or rapamycine). This study showed that matrices without drugs obtained from semiblock copolymers degraded differently than matrices containing cyclosporine A or rapamycine, whereas all types of matrices obtained from the random PLATMC degraded in a similar way. According to the outcome, a regular drug release process may be obtained from highly randomized PLATMC that remains amorphous during degradation [21]. This fact was confirmed by using poly(L-lactide-co-trimethylene carbonate) 74 : 26 with a tailored chain microstructure as a carrier for cyclosporine A (CyA). A regular degradation was determined, which also caused a uniform CyA release profile [22]. Initial studies were conducted also on poly(L-lactide-co-*ε*-caprolactone) [23]; however, more detailed analysis is needed to determine factors useful for tailoring drug release from PLACL matrices. It is especially important because of slow degradation of PCL, which makes homo- and copolymers containing caproyl units appropriate for long-term delivery. Therefore, the aim of this study was to obtain slow degradable PLACL matrices that are the most appropriate for release of immunosuppressive drugs. The copolymers with different copolymer chain microstructure were synthesized with the use of non-toxic initiator—Zr[Acac]_4_. Three kinds of poly(L-lactide-co-*ε*-caprolactone) matrices were used to compare the release process of cyclosporine A and rapamycine. They were selected according to the chain microstructure and comonomer composition. Two of them had the same composition (ratio of lactidyl to caproyl units 75 : 25) but different microstructure and the third copolymer had higher amount of caproyl units (ratio of lactidyl to caproyl units 92 : 8). The influence of copolymer chain microstructure on drug release rate and profile was analysed. 

Biodegradable matrices with immunosuppressive agent (cyclosporine A or rapamycine) could be administered locally providing sustained, prolonged release. Local immunosuppression may reduce the drug specific and general adverse consequences of systemic immunosuppression. Targeting to lymphatics has been suggested as the parameter to improve CyA formulations [24, 25]. The possibility to obtain slow release of CyA after implant administration onto the surface of thoracic duct was reported [25].

## 2. Materials and Methods

### 2.1. Synthesis of Copolymers

Three kinds of poly(L-lactide-co-*ε*-caprolactone) (PLACL) were used to prepare matrices containing cyclosporine A or rapamycine: less random 75 : 25; more random 75 : 25 and with low content of caprolactone unit 92 : 8. Copolymers with the same comonomer ratio (less random PLACL 75 : 25; more random PLACL 75 : 25) were used to compare the influence of copolymer chain microstructure on degradation and drug release profile. The third copolymer (PLACL 92 : 8) was chosen to compare the effect of copolymer composition (lower content of caprolactone units) with the influence of copolymer microstructure on drug release rate and profile. Copolymers were synthesized according to the method described in the literature [26]. Briefly, the copolymerization reactions were performed in bulk at 110°C (less random PLACL 75 : 25) or at 120°C (more random PLACL 75 : 25 and PLACL 92 : 8) in sealed glass ampoules using zirconium (IV) acetylacetonate (Zr (Acac)_4_) as non-toxic initiator with an *I*/*M* molar ratio of 1/800 (less random PLACL 75 : 25) or 1/1000 (more random PLACL 75 : 25 and PLACL 92 : 8) during 72 h. The obtained copolymers were precipitated with methanol and dried at 50°C under vacuum.

### 2.2. Characterization of the Studied Copolymers

The studied copolymers were characterized at the beginning and during the degradation process of the polymeric matrices.

The molar mass and molar mass distribution of the obtained copolymers were determined by gel permeation chromatography with a Physics SP 8800 chromatograph (tetrahydrofuran was used as the eluent, the flow rate was 1 mL/min, and Styragel columns and Shodex SE 61 detector were used). The molecular weights were calibrated with polystyrene standards.

Composition of copolymers was defined by ^1^H NMR and ^13^C NMR spectroscopy. The ^1^H NMR spectra of the studied copolymers were recorded at 600 MHz and ^13^C NMR at 125 MHz with AVANCE II Ultra Shield Plus, Bruker 600 MHz spectrometer, and a 5 mm sample tube. CDCl_3_ was used as a solvent. 

Thermal properties of polymeric matrices at the beginning and with degradation were examined by differential scanning calorimetry (DSC) with a TA DSC 2010 apparatus (TA Instruments, New Castle, DE, USA) calibrated with high purity indium and gallium. The samples were scanned from about –20°C to 220°C at a heating rate of 20°C/min. To determine the glass-transition temperature (*T*_*g*_), the samples were heated at 220°C, cooled rapidly to −20°C, and then reheated at 20°C/min to 220°C.

### 2.3. Preparation of Matrices Containing Cyclosporine A or Rapamycine

Three kinds of matrices were prepared from each kind of copolymer: matrices with 10% of cyclosporine A, 10% of rapamycine, and without drug. Matrices without drug were prepared by solution of each kind of copolymer in methylene chloride (Aldrich). In case of matrices with drug, solution of each kind of copolymer in methylene chloride was mixed with solution of 10 weight-% of studied immunosuppressive agent-cyclosporine A (CyA) or rapamycine (sirolimus) (LC Laboratories, USA) in methylene chloride. The solution was cast by means of a standard casting device on a glass plate and evaporated at ambient temperature. Then, the films were dried under reduced pressure and cut to obtain 1.2 cm diameter matrices with 0.5 mm thickness. The drug concentration in matrices (ca. 10% of copolymer content) was confirmed by means of UV-vis spectrometry.

### 2.4. *In Vitro* Studies of Cyclosporine A and Rapamycin Release from Polymeric Matrices

The weighted samples of the polymeric matrices (about 35 mg) were immersed in PBS (phosphate buffer saline; pH 7.4). The vials were incubated at 37°C under constant agitation (orbital shaker IKA KS 130basic). Each sample was prepared in triplicate. Every third or fourth day, the phosphate buffer saline was changed and drug concentration was determined in collected samples. Complete replacement of phosphate buffer saline two times a week prevented pH changes of solutions that could result from increase of degradation products. PBS was replaced also in vials containing matrices without drug. After 14, 35, 70, and 182 days of the experiment, one sample was withdrawn to assess the degradation process in polymeric matrix.

The concentration of drug released from polymeric matrices during 227 days of *in vitro* study was determined by means of UV-VIS spectroscopy (Spectrophotometer V-570, UV-VIS-NIR-JASCO). According to the literature data for cyclosporine A, the absorbance was measured at 202 nm [27] and for rapamycine (sirolimus) at 276 nm [28]. The concentration was estimated as the average of three samples. Buffer obtained from vial with matrices without drug was used as reference. PBS was changed at the same periodic intervals in all kinds of samples matrices with drug and matrices without drug. 

The presence of the studied pharmaceuticals in the polymeric matrices was confirmed by analysing the resonance signals of drug in NMR spectra, according to the literature data [29, 30] and spectrum obtained for pure drug. 

### 2.5. Microstructure Characterization of Poly(L-lactide-co-*ε*-caprolactone) during Degradation Process of the Matrices with Drug

The characterization of copolymers microstructure during degradation process (after 14, 35, 70, and 182 days) was conducted based on the parameters determined from ^1^H NMR and region of carbonyl carbon atoms in ^13^C NMR spectra: percentage content of lactidyl (*F*_LL_) and caproyl (*F*_cap_) units in copolymer; the average length of lactidyl (*l*_LL_) and caproyl (*l*_cap_) blocks in copolymer chains; and randomization ratio *R* [17, 31]. 

## 3. Results and Discussion

### 3.1. Characteristic of Copolymers Used in Formation of Controlled Delivery Systems

Three different poly(L-lactide-co-*ε*-caprolactone) (PLACL) copolymers have been synthesized for preparation of matrices with immunosuppressive drugs. Zr(Acac)_4_ was used as a non-toxic initiator that allows to obtain high molecular weight copolymers. Two of the copolymers had the same comonomer molar ratio (PLACL 75 : 25, where 75 : 25 denotes molar ratios of comonomers in the copolymer determined by ^1^H NMR) but different microstructure (PLACL 75 : 25 *R* = 0.68 and PLACL 75 : 25 *R* = 0.75). Coefficient *R* is a measure of the degree of randomness of the copolymer chain. It attains value 0 for diblock copolymer and 1 for completely random distribution of lactyl and caproyl units in copolymer chain [32]. The third of the studied copolymer had lower content of caproyl units (PLACL 92 : 8).

**Figure 1:**
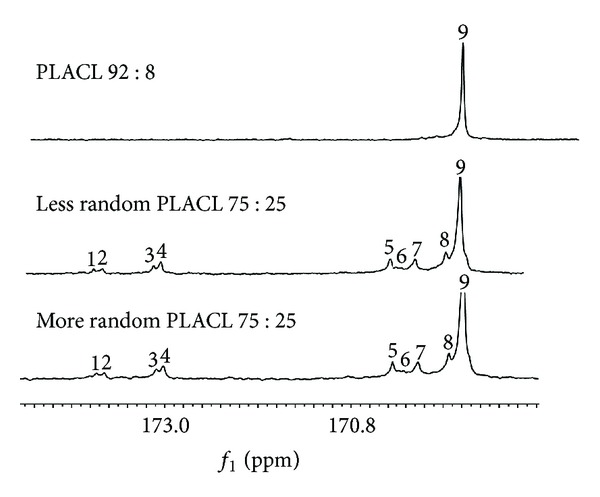
^13^C NMR spectra of matrices made of less random PLACL 75 : 25; more random PLACL 75 : 25 and PLACL 92 : 8 before degradation. Carbonyl carbon region of *ε*-oxycaproyl unit: (1) CapCapCap; (2) LLCapCap; (3) CapCapLL; (4) LLCapLL; (5) LLLLCap; (6) CapLLCap; (7) CapLLLL; (8) LLLLCap; (9) LLLLLL.

Microstructure of three kinds of poly(L-lactide-co-*ε*-caprolactone) which were used to form matrices has been analysed based on the parameters determined from NMR spectra (Table 1, Figure 1). 

**Figure 2:**
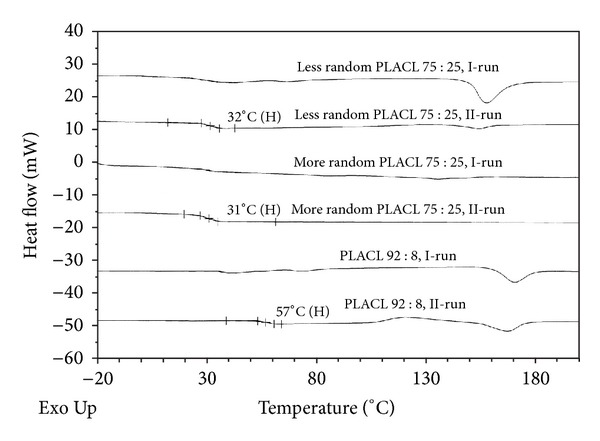
Thermal properties of three kinds of PLACL used to prepare matrices with cyclosporine A and rapamycine.

Less random PLACL 75 : 25 and more random PLACL 75 : 25 characterized segmental structure. Although the PLACL 75 : 25 has high randomization ratio, it characterizes rather segmental structure. Random copolymers contain sequences as CapLCap, which result from transesterification of the second mode that takes place during copolymerization reaction. In the case of PLACL copolymers, this reaction leads to scission of lactidyl (LL) sequences and formation of random sequences that contain also lactyl (L) units. Thus, lactyl (L) and caproyl (Cap) sequences should be taken into consideration as the structural units of the random copolymer chain. CapLCap sequence was not detected in any of the studied copolymers, which means that transesterification of the second mode did not occur [32]. Only comonomers unit content was determined for PLACL 92 : 8, because resonance lines arising from carbonyl carbon region of *ε*-oxycaproyl units were not well visible in ^13^C NMR spectrum as a result of small amount of carbonyl units (Figure 1). This copolymer had the highest molar mass (105.1 kDa). More random PLACL 75 : 25 characterized higher randomization ratio and shorter average of lactidyl and caproyl blocks (Table 1) than less random PLACL 75 : 25, which resulted from different conditions of copolymerization reaction. More random PLACL 75 : 25 was synthesized at higher temperature, which accelerates intermolecular transesterification processes. Thermal properties were analysed by means of DSC: the glass transition temperature was determined from the second run and the melting transition from the first run (Figure 2). 

The *T*_*g*_ of less random PLACL 75 : 25 and more random PLACL 75 : 25 was below 37°C (32°C and 31°C, resp.), which means that in conditions of the experiment they were in elastic state. More random PLACL 75 : 25 exhibited only the glass transition temperature. Very low melting enthalpy at a second run was detectable in case of less random PLACL 75 : 25. Significantly higher *T*_*g*_ was determined for PLACL 92 : 8 (56°C). 

### 3.2. Release Profiles of Immunosuppressive Drugs and Changes in Matrices Microstructure during Degradation

**Figure 3:**
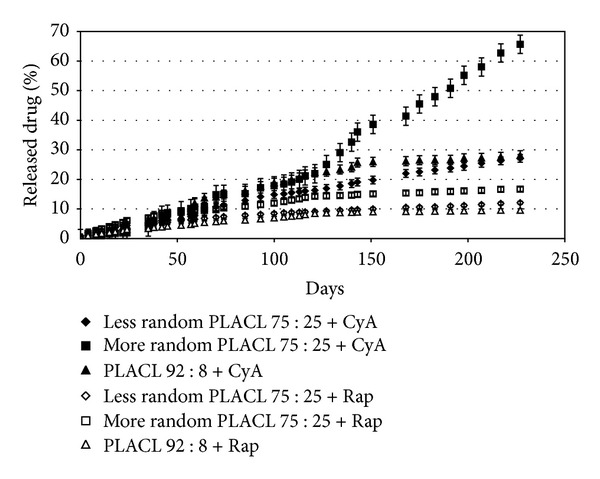
Cumulative release of cyclosporine A and rapamycine from PLACL matrices during 227 days. Each point represents the mean ± SD of three points.

**Figure 4:**
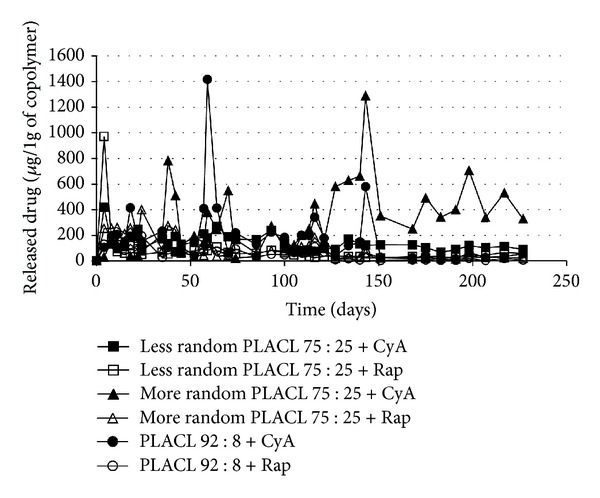
The release profile of cyclosporine A and rapamycine from different kinds of PLACL matrices during 227 days (*μ*g/1 g of copolymer). Each point represents the mean of three points.

Detailed analysis of drug release process (Figures 3 and 4) and changes in copolymer chain microstructure during degradation of three kinds of poly(L-lactide-co-*ε*-caprolactone) matrices (less random 75 : 25; more random 75 : 25 and 92 : 8) containing CyA or rapamycine has been conducted. High drug loading efficiency (above 80%) was obtained for both CyA and rapamycine matrices. 

#### 3.2.1. Matrices with Immunosuppressive Drug Obtained from Less Random PLACL 75 : 25

*Changes of Copolymer Chain Microstructure during Degradation. *The details about changes in copolymer chain microstructure during 182 days of degradation are presented in Table 2. 

**Figure 5:**
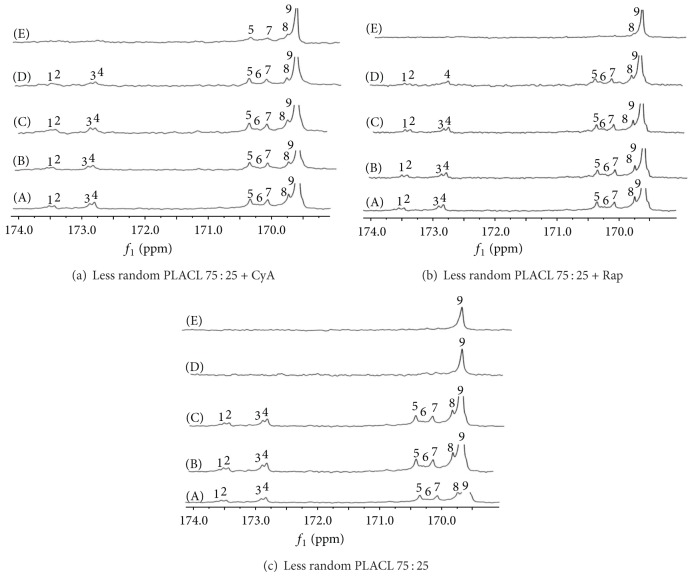
^13^C NMR spectra of matrices made of less random PLACL 75 : 25: (a) with CyA; (b) with rapamycine; (c) without drug, before degradation (A) and after 14 (B), 35 (C), 70 (D) and 182 (E) days of degradation. Carbonyl carbon region of *ε*-oxycaproyl unit: (1) CapCapCap; (2) LLCapCap; (3) CapCapLL; (4) LLCapLL; and carbonyl carbon region of lactidyl unit (5) LLLLCap; (6) CapLLCap; (7) CapLLLL; (8) LLLLCap; (9) LLLLLL.

The comparison of polymer chain microstructure of matrices containing immunosuppressive agent and drug-free matrices showed that their degradation processes proceeded differently (Table 2, nos. 1–15). In drug-free matrices, caproyl blocks underwent degradation first—only 3% of caproyl units remained in polymeric material after 182 days (Table 2, no. 5). In case of matrices with cyclosporine A and with rapamycine, caproyl units degraded less rapidly (16% of caproyl units remained after 182 days) (Table 2, nos. 10 and 15). Therefore, caproyl units degraded faster in matrices without drug than in matrices with pharmaceutical agent (which characterized more significant decrease of caproyl units after 182 days). Apparently, in matrices without drug, randomized sequences of CapLLCap type degraded firstly. Gradual increase of lactidyl units' content (Table 2, nos. 1–5) could have led to decrease of randomization and arrangement of homopolilactidyl blocks, which caused crystallization and slower degradation process. The melting enthalpy of crystallite domains was already determined in thermogram of less random PLACL 75 : 25 before degradation (Figure 2). In case of matrices with CyA or rapamycine, uniform removal of caproyl and lactidyl units was determined until day 70 (Figure 5), as a result of regular drug distribution in matrix, which prevented the forming of crystalline domains.

In the case of matrices with cyclosporine A, insignificant changes in copolymer chain microstructure were observed during 70 days (Table 2, nos. 6–9)—a slight decrease of caproyl units content and increase of the average length of lactidyl sequences. Increase of the lactidyl units in copolymer and the average length of the lactidyl sequences were observed between day 70 and 182 (Table 2, no. 9-10). Slight decrease of randomization ratio was also observed. In the case of less random PLACL 75 : 25 with rapamycine (Table 2, nos. 11–15), constant decrease of the average length of lactidyl and caproyl sequences was determined until day 70 (Table 2, nos. 11–14). More significant decrease of caproyl units was observed between day 70 and 182 (Table 2, nos. 14-15), which was similar to degradation process observed in matrices with cyclosporine A. Changes in copolymer chain microstructure detected in carbonyl region are shown in the spectra presented in Figure 5(b). All resonance signals arising from carbonyl carbons of caproyl and lactidyl groups are present in the spectra obtained after 14 and 35 days of degradation. After 182 days of degradation process, signals at 172.8–173.5 ppm were not observed. Very small changes in randomization ratio were noticed during the whole process.

*Drug Release Study from Less Random PLACL 75 : 25. *A burst effect was observed in case of matrices with cyclosporine A (Figure 4), which caused release of 418 *μ*g of CyA per 1 g of copolymer. Then, as a result of insignificant changes in copolymer chain microstructure (Table 2, nos. 6–9), the drug release process proceeded evenly with the average amount of released cyclosporine of 114.6 *μ*g/1 g of copolymer until day 70 and 124.5 *μ*g/1 g of copolymer between day 70 and 182. 

**Figure 6:**
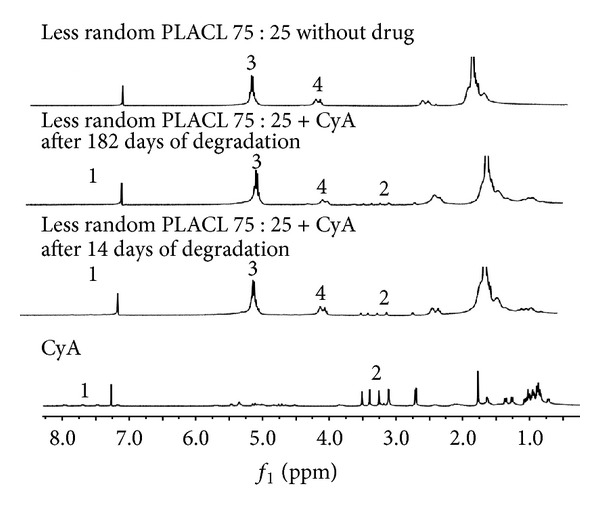
^1^H NMR spectra of CyA in CDCl_3_; matrix made of less random PLACL 75 : 25 with CyA (after 14 and 182 days of degradation); matrix made of less random PLACL 75 : 25 without drug. Assigned signals arise from cyclosporine A: NH (1) and N–CH_3_ (2); methine groups of lactidyl units (3) and OCH_2_ groups of caproyl unit of copolymer (4).

Degradation process of less random PLACL 75 : 25 matrices did not cause any changes in drug molecule–the proper structure of CyA was observed in ^1^H NMR spectrum.  Figure 6 presents comparison of proton spectra of pure CyA, less random PLACL 75 : 25 matrix without drug, and less random PLACL 75 : 25 matrices with CyA after 14 and 182 days of degradation. Resonance lines of CyA groups: NH (1) and N–CH_3_ (2) are shown. 

The release profile of rapamycine from less random PLACL 75 : 25 characterized burst effect (970.2 *μ*g/1 g of copolymer) (Figure 4) followed by slow and even release with the average of 51.4 *μ*g/1 g of copolymer. 

#### 3.2.2. Matrices with Immunosuppressive Drug Obtained from More Random PLACL 75 : 25

*Degradation of More Random PLACL 75 : 25. *Degradation process of more random PLACL 75 : 25 matrices with cyclosporine A, rapamycine, and drug-free matrices proceeded in the same way (Table 3, nos. 1–15). Gradual decrease of caproyl units and increase of lactidyl units' content were observed in case of the three kinds of matrices. 

Copolymerization reaction of more random PLACL 75 : 25 was conducted at higher temperature (120°C) that enabled the forming of short, random sequences of CapLLCap, LCapL type, resulting in amorphous structure of copolymer (Figure 2). This structure of copolymer chain may be the reason of similar degradation of matrices with and without drug. However, significant increase of lactidyl units was observed between days 105 and 210 that probably led to arrangement of homopolilactidyl sequences into crystalline domains. It is known that morphology of a polymeric material plays a pivotal role in degradation process. Degradation of semicrystalline polyesters in aqueous media occurs in two stages. During the first stage, water diffuses into amorphous regions and causes random hydrolytic scission of ester bonds. After preferential degradation of amorphous regions, in the second stage, water penetrates into crystalline domains; however, degradation rate is much slower in crystallites [1]. Increase of lactidyl units' content had different consequences in case of matrices with CyA and matrices with rapamycine, as will be presented below.

*Drug Release from More Random PLACL 75 : 25. *Matrices obtained from more random PLACL 75 : 25 with cyclosporine A characterized regular drug release process at the beginning, at an average of 102.1 *μ*g/1 g of polymer (Figure 4). Burst of drug was not observed (Figures 3 and 4); however, the amount of released CyA increased to 782.2 *μ*g/1 g of copolymer at day 38 and then decreased to about 306.1 *μ*g/1 g of copolymer (between days 70 and 105). Significantly more drug was released between days 105 and 210 (at an average of 467.2 *μ*g/1 g of copolymer). The highest amount of released cyclosporine A was 1288.3 *μ*g/1 g of copolymer. The effect of higher drug release was observed for all studied samples. Thus, it can be concluded that the three effects of significantly higher CyA release (two between days 35 and 70 and one between day 105 and 210) (Figure 4) may have been caused by increase of randomization ratio and decrease of the average length of lactidyl units. Release of longer copolymer blocks may cause also rapid drug release.

The release profile of rapamycine was different than that of CyA. It was observed that during 105 days, the amount of released drug was higher (about 166 *μ*g/1 g of copolymer), then decreased to about 51.6 *μ*g/1 g of copolymer (between days 105 and 210). It should be mentioned that this process proceeded evenly and the burst effect was not observed, which was similar to matrices with CyA (Figures 3 and 4). 

The lack of burst effect in case of both kinds of matrices (containing CyA or rapamycine) was probably caused by uniform distribution of drug molecules in matrix obtained from copolymer with large amount of random sequences. However, differences in further release process were determined despite similar degradation of matrices. As was mentioned before, significant increase of lactidyl units' content between days 105 and 210 had different consequences in case of matrices with different drugs. After day 105, more CyA was released than rapamycine. This may be explained as a result of releasing over 18% of cyclosporine A until day 105 (whereas only 12% of rapamycine was released) that probably formed voids between copolymer chains in matrix. The tunnels formed in matrix may cause diffusion of drug molecules outside and accelerate hydrolysis by allowing easier water absorption inside [33].

#### 3.2.3. Matrices with Immunosuppressive Drug Obtained from Copolymer with Low Content of Caprolactone Unit—PLACL 92 : 8

*Degradation of PLACL 92 : 8. *As was mentioned before, only the comonomer units' ratio was determined during degradation process in case of PLACL 92 : 8 matrices. The lactidyl to caproyl unites ratio at the beginning, after 14, 35, 70, and 182 days, for PLACL 92 : 8 matrices with CyA was, respectively; 92 : 8, 94 : 6, 94 : 6, 94 : 6, 95 : 5; for matrices with rapamycine 92 : 8, 93 : 7, 94 : 6, 93 : 7, 93 : 7; for matrices without drug 92 : 8, 92 : 8, 94 : 6, 94 : 6, 95 : 5.

*Drug Release Profile of PLACL 92 : 8. *Cyclosporine A was released from PLACL 92 : 8 matrices evenly from the beginning, without burst effect (Figures 3 and 4), at an average of 204.6 *μ*g/1 g of copolymer during 35 days, 322.7 *μ*g/1 g of copolymer from day 35 to 70 and 164.5 *μ*g/1 g of copolymer between day 70, and 105 (Figure 4). Higher amount of released drug was detected at day 59 (1416.5 *μ*g/1 g of copolymer). The average amount of released drug at the last phase (between days 105 and 210) was 94.7 *μ*g/1 g of copolymer (except from the increase detected at day 143 : 578.6 *μ*g/1 g of polymer). 

Rapamycine was released from PLACL 92 : 8 matrices very evenly, at an average of 131 *μ*g/1 g of copolymer during 55 days, 86.3 *μ*g/1 g of copolymer between days 35 and 70; 57.2 *μ*g/1 g of copolymer between days 70 and 105, and 31.5 *μ*g/1 g of copolymer from day 105 to 210. The amount of released drug was decreasing with time, but the process proceeded very evenly (Figures 3 and 4). 

### 3.3. Comparison of Cyclosporine A and Rapamycine Release Profile from Three Kinds of PLACL Matrices

**Figure 7:**
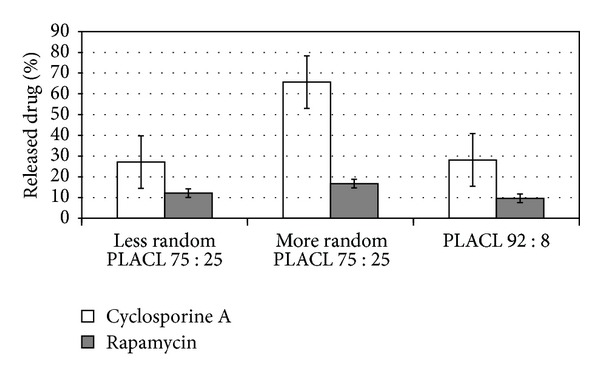
Comparison of the amount of released immunosuppressive drugs from three kinds of poly(L-lactide-co-*ε*-caprolactone) matrices after 227 days. Each point represents the mean ± SD of three points.

In case of all studied PLACL matrices, long-term degradation and drug release were observed. The statistically significant differences were determined between more random PLACL 75 : 25 and two other copolymers—after 227 days, the highest amount of both studied drugs was released from more random PLACL 75 : 25 (66.7% of CyA and 16.7% of rapamycine). Moreover, the amount of cyclosporine A and rapamycine released from three kinds of poly(L-lactide-co-*ε*-caprolactone) matrices with different copolymer chain microstructure was compared (Figure 7). 

Despite the same comonomers content of less random PLACL and more random PLACL, similar molar mass (resp.: 60, 300, and 63 500 Da), they revealed completely different drug release rates and profiles (e.g., the burst effect of both drugs was observed in case of less random PLACL 75 : 25 but was not noticed in more random PLACL 75 : 25). This suggests the influence of copolymer chain microstructure on drug release process. Apparently, more randomized structure of more random PLACL 75 : 25 was prevented from crystallization during degradation process and accelerated the drug release rate. Less random PLACL 75 : 25 characterizes longer lactidyl units at the beginning (Table 1) and increase of their length during degradation (Table 2, nos. 5, 10, and 15). The thermogram of less random PLACL 75 : 25 (Figure 2) shows the presence of crystalline domains in this copolymer. Similar amount of both drugs was released from less random PLACL 75 : 25 and PLACL 92 : 8 (resp.: 29.9% and 28.1% in case of CyA and 12.2% and 9.6% in case of rapamycine), which was not expected because PLACL 92 : 8 had much higher molar mass than less random PLACL 75 : 25 and higher *T*_*g*_, which was above 37°C (temperature of *in vitro* degradation) (Table 1, Figure 2). This evidences that those differences and copolymer composition (e.g., lower amount of caprolactone units) do not have to have an impact on drug release profile. The dominant is the influence of copolymer microstructure. PLACL 92 : 8 contained higher amount of lactidyl units, however, was synthesized at higher temperature than less random PLACL 75 : 25 that promotes arising more randomized structure of copolymer chain, which decreases the susceptibility for crystallization. 

The release profile of rapamycine from all of the studied PLACL matrices characterized lack of uncontrolled burst effects; however, the amount of released drug was maintained at very low level in case of less random PLACL 75 : 25 and PLACL 92 : 8 (Figures 3 and 4). Random structure of copolymer accelerated the release rate of rapamycine. The most even CyA release was observed from less random PLACL 75 : 25 and PLACL 92 : 8 (Figures 3 and 4). In case of more random PLACL 75 : 25, higher molar mass dispersity (*D* = 2.5) (Table 1) indicates higher amount of shorter copolymer chains, in which faster release might have caused uneven drug release. In our opinion PLACL with uniform molecular mass dispersion and more random structure (*R* ≈ 1) would provide regular release process of both cyclosporine A and rapamycine. This fact was also confirmed in another study on matrices obtained from poly(L-lactide-co-trimethylene carbonate) (PLATMC), where a similar effect was observed. Similar amounts of released drugs were determined for PLATMC 28 : 72 (*R* = 0.57) and PLATMC 72 : 28 (*R* = 0.5) despite the different copolymer compositions. The highest amount of cyclosporine A was released from more random PLATMC 72 : 28 (*R* = 0.85), but also more fluctuations in drug release profile were noted [22]. Therefore, it may be concluded that regardless of copolymer composition, steady drug release process may be obtained from highly randomized copolymers (*R* ≈ 1) and narrow molar mass dispersity. 

Cyclosporine A was released significantly faster than rapamycine from each kind of the studied copolymer. According to some authors, CyA, which contains secondary and tertiary nitrogen atoms and a hydroxylic group in aminoacid 1 may accelerate hydrolysis of polyester [34]. 

Different degradation process was determined in case of less random PLACL 75 : 25 and more random PLACL 75 : 25. It was determined that in case of matrices obtained from less random PLACL 75 : 25 without drug, mainly caproyl units underwent degradation. The increase of lactidyl units' content during degradation of PLACL and selection of lactyl-rich crystalline residues relatively more resistant to degradation was already described [31]. Incorporation of drug molecule into the matrix delayed this effect, because in case of the same kind of copolymeric matrices containing immunosuppressive drug, the decrease of caproyl unit content was not observed until day 70. In case of all kinds of more random PLACL 75 : 25 matrices (Table 3) and PLACL 92 : 8, the compositional changes were almost the same. Taking into account different conditions of copolymerization reaction of less random PLACL 75 : 25 (temperature of reaction: 110°C and *I*/*M* = 1/800) and more random PLACL 75 : 25 (temperature of reaction: 120°C and *I*/*M* = 1/1000), sequences of CapLLCap, LCapL type are expected in case of more random PLACL 75 : 25 and PLACL 92 : 8. These kinds of units, as was mentioned above, determine regular degradation and less influence drug molecule on this process. 

The conducted research indicates the advantages of applying biodegradable polymers with immunosuppressive agents, which can provide long-term release, desired for these kinds of drugs. Although some attempts to develop biodegradable carriers of immunosuppressants have already been described, none of them concerned prolonged delivery. However, prediction of drug release kinetics from biodegradable polymeric matrices is very difficult and complicated because of two simultaneous processes—polymer erosion and drug diffusion through performed microporous channels within the matrices. Both degradation of drug delivery devices and drug release profile are influenced by factors as morphology, crystallinity of the polymer, formulation, drug molecular size, and water solubility [35]. This may explain the results of this study. Different drug release profiles have been obtained depending not only on different copolymers but also on the kind of drug released from the same kind of copolymer. This may result from the size and structure of drug molecule (molecular weight for CyA is 1202.64 and 914.19 for sirolimus); differences in number and type of functional groups can react by hydrogen bonds with polyester material and influence hydrophobicity of the whole drug-polymer system. However, all of the drug release profiles were strongly associated with the process of polymer degradation. 

In case of CyA, some authors described biodegradable nanoparticles and microspheres as unstable systems, characterized by biphasic release patterns—rapid initial release of CyA and slower release thereafter [33]. In the present study, the polymeric matrices were chosen as an optimal form to study copolymer chain microstructure. The goal of the presented research was to analyze the correlation between drug release profile and degradation process determined by polymeric chain microstructure and only simple release system allows observing both of them. The obtained results may be helpful for developing a method to control the rate of drug release by using polymeric carrier with particularly designed microstructure. This is the best form to study materials that may be used as immunosuppressants releasing implantable systems, but also coatings for metallic or biodegradable stents. Both studied drugs, cyclosporine A and rapamycine, demonstrate inhibitory effect on proliferation of smooth muscle cells, so they are considered in prevention of in-stent restenosis [36, 37]. Taking into account the subject of this study, the drug amount incorporated into the polymeric material was not concerned as a therapeutical dose. In some cases, as for rapamycine eluting biodegradable stents, low (64 *μ*g/mL/stent) or high dose (196 *μ*g/mL/stent) is taken into account. It is known that the inhibition of the vascular smooth muscle cells growth is concentration dependent, with the threshold limit of 16.7 ng/mL [13]. The blood level of CyA in transplant patient should be maintained in the range of 150–300 ng/mL [11, 28]. In ophthalmologic applications, CyA concentrations of 25–75 *μ*g/mL were measured in human tears after oral daily administration of 5 mg/kg [38]. The final concentration of rapamycine in the present study was about 3.2 *μ*M and 2.4 *μ*M of CyA. Even as low concentration of cyclosporine A as 120 nM was proved to inhibit lymphocyte proliferation [11]. The results of our study may be helpful in developing systems, which can provide release of very low sustained doses of these drugs during several months after organ transplantation and in formation of coating films on nondegraded metallic and plastic implants.

## 4. Conclusions

In case of all of the studied poly(L-lactide-co-*ε*-caprolactone) matrices (less random 75 : 25, more random 75 : 25 and 92 : 8), slow degradation process was observed which make them appropriate for cyclosporine A and rapamycine delivery. However, it was observed, that drug release profiles were dependent on the kind of copolymer. Moreover, various chemical structures of cyclosporine A and rapamycine caused differences in drug release even from the same kind of copolymer matrix. The highest amount of both studied drugs was released from more random PLACL 75 : 25. Despite the same comonomers content of less random PLACL and more random PLACL, they revealed different drug release profiles (e.g., the burst effect was observed only in less random PLACL 75 : 25) and rate. This suggests the influence of copolymer chain microstructure on drug release process. Moreover, similar amount of both drugs was released from less random PLACL 75 : 25 and PLACL 92 : 8. This evidences that different copolymer composition (e.g., lower amount of caprolactone units) does not have to have an impact on drug release profile. The dominant is the influence of copolymer microstructure. 

It is difficult to tailor the kinetics of drug release because of many factors that affect this process; however, copolymer chain microstructure should be taken into account as an important parameter that influences copolymer degradation and drug release process. The correlation between drug release and changes of copolymer microstructure during degradation was observed. Based on the obtained results, we can conclude that highly randomized poly(L-lactide-co-*ε*-caprolactone) with uniform molar mass dispersion should provide regular release of cyclosporine A and rapamycine.

It is important that this fact was confirmed also in another study on matrices obtained from poly(L-lactide-co-trimethylene carbonate) (PLATMC), where a similar effect was observed. Similar amounts of released drugs were determined for PLATMC 28 : 72 (*R* = 0.57) and PLATMC 72 : 28 (*R* = 0.5) despite the different copolymer compositions. The highest amount of cyclosporine A was released from more random PLATMC 72 : 28 (*R* = 0.85), but also more fluctuations in drug release profile were noted [22]. Therefore, it may be concluded that regardless of copolymer composition, steady drug release process may be obtained from highly randomized copolymers (*R* ≈ 1) and narrow molecular mass dispersion. Thus, the possibility to tailor microstructure of both poly(l-lactide-co-*ε*-caprolactone) and poly(L-lactide-co-trimethylene carbonate) (PLATMC) obtained with the use of Zr(Acac) as the initiator of copolymerization reaction and confirmed biocompatibility of similar polymers obtained with the same initiator [39, 40] make them promising candidates for developing delivery systems of immunosuppressive drugs.

## Figures and Tables

**Table 1:** Characterization of poly(L-lactide-co-*ε*-caprolactone) matrices containing cyclosporine A or rapamycine (*M*_*n*_: number-average molar mass; *D*: molar-mass dispersity; *T*_*g*_: glass transition temperature, *T*_*m*_: melting temperature; *l*_LL_, *l*_Cap_: the average length of lactidyl and caproyl blocks; *R*: randomization ratio; *I*/*M*: initiator to monomer molar ratio).

Copolymer	*M*_*n*_ (Da)	*D *	*T*_*g*_^a^ (°C)	*T*_*m*_^b^ (°C)	Average length of blocks	*R *	Copolymerization conditions		
							*I*/*M*	Temp.	Time
Less random PLACL 75 : 25	60 300	2.1	32	158	*l*_LL_ = 5.13 *l*_Cap_ = 1.71	0.68	1/800	110°C	72 h
More random PLACL 75 : 25	63 450	2.5	31	—	*l*_LL_ = 4.28 *l*_Cap_ = 1.58	0.75	1/1000	120°C	72 h
PLACL 92 : 8	105 100	2.2	57	170	—	—	1/1000	120°C	72 h

^a^Obtained by DCS (second heating); ^b^obtained by DSC (first heating).

**Table 2:** Microstructure of less random PLACL 75:25 matrices containing cyclosporine A or rapamycine (*F*_LL_, *F*_Cap_: percentage molar content of lactidyl and caproyl unit; *l*_LL_, *l*_Cap_: the average length of lactidyl and caproyl sequences; *R*: randomization ratio) after 14, 35, 70, and 182 days of degradation.

Matrix	No.	days	*F*_LL_	*F*_Cap_	*l*_LL_	*l*_Cap_	*R *
Less random PLACL 75 : 25	1	0	75	25	5.13	1.71	0.68
	2	14	75	25	5.17	1.72	0.68
	3	35	76	24	5.07	1.60	0.72
	4	70	92	8	—	—	—
	5	182	97	3	—	—	—

Less random PLACL 75 : 25 + CyA	6	0	75	25	5.13	1.71	0.68
	7	14	75	25	5.64	1.65	0.67
	8	35	76	24	6.05	1.64	0.60
	9	70	76	24	6.04	1.60	0.60
	10	182	84	16	10.65	2.03	0.54

Less random PLACL 75 : 25 + Rap	11	0	75	25	5.13	1.71	0.68
	12	14	75	25	5.87	1.71	0.65
	13	35	75	25	4.92	1.64	0.70
	14	70	75	25	4.80	1.60	0.73
	15	182	84	16	12.7	1.60	0.67

**Table 3:** Microstructure of more random PLACL 75:25 matrices containing cyclosporine A or rapamycine (*F*_LL_, *F*_Cap_: percentage molar content of lactidyl and caproyl unit; *l*_LL_, *l*_Cap_: the average length of lactidyl and caproyl sequences; *R*: randomization ratio) after 35, 70, 105, and 210 days of degradation.

Matrix	No.	Days	*F*_LL_	*F*_Cap_	*l*_LL_	*l*_Cap_	*R *
More random PLACL 75 : 25	1	0	75	25	4.28	1.58	0.75
	2	35	74	26	4.02	1.43	0.81
	3	70	74	26	4.00	1.41	0.83
	4	105	78	22	5.72	1.73	0.68
	5	210	82	18	6.67	1.47	0.74

More random PLACL 75 : 25 + CyA	6	0	75	25	4.28	1.58	0.75
	7	35	73	27	4.03	1.45	0.81
	8	70	73	27	3.66	1.32	0.89
	9	105	75	25	6.23	2.05	0.57
	10	210	81	19	6.37	1.5	0.74

More random PLACL 75 : 25 + Rap	11	0	75	25	4.28	1.58	0.75
	12	35	73	27	3.99	1.44	0.81
	13	70	73	27	4.39	1.58	0.74
	14	105	75	25	5.28	1.76	0.66
	15	210	82	18	6.68	1.47	0.76
